# Fungal gut microbiota dysbiosis in systemic lupus erythematosus

**DOI:** 10.3389/fmicb.2023.1149311

**Published:** 2023-04-05

**Authors:** Ping Yang, Rui Xu, Fei Chen, Shanshan Chen, Adeel Khan, Liang Li, Xiaoshan Zhang, Yanbo Wang, Zhipeng Xu, Han Shen

**Affiliations:** ^1^Department of Clinical Laboratory, The Affiliated Drum Tower Hospital of Nanjing University Medical School, Nanjing, Jiangsu, China; ^2^State Key Laboratory of Pharmaceutical Biotechnology, Department of Physiology, Jiangsu Engineering Research Center for MicroRNA Biology and Biotechnology, School of Life Sciences, NJU Advanced Institute of Life Sciences (NAILS), Nanjing University, Nanjing, China; ^3^Department of Rheumatology and Immunology, Affiliated Nanjing Drum Tower Hospital, Medical School of Nanjing University, Nanjing, Jiangsu, China; ^4^Department of Biotechnology, University of Science and Technology, Bannu, Pakistan; ^5^Department of Pathogen Biology, Jiangsu Province Key Laboratory of Modern Pathogen Biology, Nanjing Medical University, Nanjing, Jiangsu, China

**Keywords:** systemic lupus erythematosus, lupus nephritis, fungal microbiota, ITS, biomarkers

## Abstract

**Introduction:**

Despite recent developments in our comprehension of how the gut microbiota and systemic lupus erythematosus (SLE) are related. The mycobiome: which is a small but crucial part of the gut microbiota and is involved in hosts’ homeostasis and physiological processes, remained unexplored in SLE.

**Methods:**

We profiled the gut fungal mycobiota based on internal transcribed spacer region 1 (ITS1) sequencing for the gut microbial DNA from the SLE individuals with lupus nephritis (LN) (*n* = 23), SLE without LN (*n* = 26) and healthy controls (*n* = 14) enrolled in Nanjing Drum Tower Hospital, The Affiliated Hospital of Nanjing University Medical School.

**Results:**

The ITS sequencing generated a total of 4.63 million valid tags which were stratified into 4,488 operational taxonomic units (OTUs) and identified about 13 phyla and 262 genera. Patients with SLE were characterized with unique fungal flora feature. The fungal microbiomes of the three groups displayed distinct beta diversity from each other. Compared with HC group, the abundance of fungal dysbiosis was reflected in a higher ratio of opportunistic fungi in SLE or LN group, as well as the loss of *Rhizopus* and *Malassezia*. The main principal components of the flora between the SLE and LN group were generally consistent. The relative abundance of *Vanrija* in the fecal fungal community was higher in LN group, while the relative abundance of *Fusarium* was higher in SLE group. Moreover, our data revealed superior diagnostic accuracy for SLE with the fungal species (e.g. *Candida*, *Meyerozyma*). Correlations between gut fungi and clinical parameters were identified by Spearman’s correlation analysis. Interestingly, *Aspergillus* in SLE patients was positively correlated with ACR, 24 h proteinuria, proteinuria, anti-dsDNA, ANA, and SLEDAI, while *Rhizopus* was negatively correlated with lymphocytes and Hb. Finally, we successfully cultured the fungi and identified it as *Candida glabrata* by microscopic observation and mass spectrometry.

**Discussion:**

We first explored the highly significant gut fungal dysbiosis and ecology in patients with SLE, and demonstrated the applicability of fungal species as SLE diagnostic tools, signifying that the gut fungal mycobiome-host interplay can potentially contribute in disease pathogenesis.

## 1. Introduction

An autoimmune disease affecting multiple organs called systemic lupus erythematosus (SLE) is driven by overactivation of host defense system and causing immunological response to the simplest components of life (Azzouz et al., 2019; Silverman, 2019). The heterogeneity of disease manifestations, multi-organ involvement in different individuals, as well as the unpredictable dynamic variability of disease activity from remission to aggravation and overall progression, present clinical challenges for SLE diagnosis and effective management. Indeed, such heterogeneity suggests that the symptoms of SLE are highly complex and unknown, not just a single phenotype (Azzouz et al., 2019). Serum autoantibodies are considered as a specific diagnostic criterion for SLE (Sharp et al., 1971), and a prognostic factor for the development of lupus nephritis (LN); which affects 30–60% of patients (Yang et al., 2021). According to investigations, fungi may contribute to the onset of autoimmune disorders like rheumatoid arthritis (RA) and inflammatory bowel disease (IBD) (Sokol et al., 2017; Iliev, 2022; Lee et al., 2022). It is speculated that initially, a primary infection triggers or induce the autoimmune response (Sanderson et al., 2017). However, the gut fungal microbiome analysis in these processes has not been explored to a large extent, resulting in the lack of potential ways to develop new SLE screening and treatment methods. For the diagnosis and therapy of SLE to be successful, a deeper understanding of the pathophysiological roots of the disease may be necessary.

Growing data suggests that particular molecular patterns associated with gut microbiota dysbiosis contribute to the emergence of SLE (Citi, 2018; Azzouz et al., 2019; Guo et al., 2020; He et al., 2020; Chen B. et al., 2021; Tomofuji et al., 2021; Chen et al., 2022; Shirakashi et al., 2022). *Streptococcus anginosus* and *Streptococcus intermedius* were discovered to be rising in SLE conditions and can play roles in the pathogenesis of SLE (Tomofuji et al., 2021). Moreover, in taxonomic diversity, the patients with higher Systemic Lupus Erythematosus Disease Activity Index (SLEDAI) scores had more significant restrictions (Azzouz et al., 2019). What is more exciting, a randomized, double-blind, placebo-controlled, multicenter clinical study provides sufficient evidence for evaluating the long-term safety and effectiveness of fecal microbiota transplantation (FMT) in SLE patients (Huang et al., 2022). Co-colonization of bacteria and fungi in the mammalian gastrointestinal tract, respiratory tract, skin epithelium, and reproductive organs form a complex ecosystem of microorganisms-microorganisms interactions and host-microorganisms interactions. These interactions are vital for human health and for understanding the pathogenesis of various human ailments (Dohlman et al., 2022). In the shadow of a bacterial resistance pandemic, fungal infections are increasing and resistance to treatment is rising developing into a public health problem worldwide (Bongomin et al., 2017; Lionakis and Levitz, 2018). There exists a scarcity of research related to the understanding of fungi’s symbiotic relationships with the immune system (Ost et al., 2021). Research on fungal communities has advanced much more slowly than that on bacterial communities due to the lower abundance and lack of a comprehensive reference genome database. Fungi can affect host immunity and infect populations with impaired immune systems (Wang et al., 2022). They are major commensal/opportunistic pathogens (Doron et al., 2021; Leonardi et al., 2022). The current research on fungi focuses on cancer patients, including cancer-associated gut fungi (Alam et al., 2022; Lin et al., 2022) and cancer- type intracellular fungi (Dohlman et al., 2022; Narunsky-Haziza et al., 2022; Yang et al., 2022), but little research is known about their role in autoimmune diseases, especially in SLE (Chen et al., 2012; Chen Y. et al., 2021; Su et al., 2021). Therefore, it is essential to characterize the fungal microbiome in SLE patients in order to comprehend the potential functions and mechanisms of fungi in the onset and progression of SLE.

In this research, we carried out internal transcribed spacer region 1 (ITS1) gene sequencing, which has shown particular promise for the molecular identification of fungi (Yuan et al., 2018; Ahmed et al., 2022), to investigate the detailed profile of the gut fungal microbiome of patients with SLE. These results may help in the development of improved SLE prevention and treatment methods. To the best of our knowledge, this study is the first of its type to adequately demonstrate the relationship between the gut fungi microbiome and clinical parameters, especially to establish the potential diagnostic and therapeutic utility of fungi in SLE.

## 2. Results

### 2.1. Basic information of study participants

Intestinal fungal profiling was performed from fecal samples collected from 49 patients with SLE and 14 healthy control (HC) volunteers in the Affiliated Drum Tower Hospital of Nanjing University Medical School from January 2022 to July 2022. For the purpose of comparison, the samples were split into three groups: 14 HC, 26 SLE patients without LN, and 23 SLE patients with LN. The detailed clinical characteristics are shown in Table 1. Age, gender, and a few other immunological indications are included in the participant information. There were no appreciable variations in ages or sex between the SLE and HC groups. However, there were significant differences in proteinuria, hematuria, pyuria, 24 h proteinuria, ACR, blood albumin, EGFR, and SLEDAI in SLE with or without LN group (*p* < 0.001). None of the patients involved in the research got antifungal treatment prior to enrolment.

**TABLE 1:** Clinical characteristics of the studied groups of patients and healthy individuals.

Clinical characteristic	HCs (*n* = 15)	SLE without LN (*n* = 26)	SLE with LN (*n* = 23)	*p*-value (SLE without LN vs. SLE with LN)
Age (years)	40.0 (25.0–45.0)	37.0 (28.8–47.0)	35.0 (23.0–44.0)	0.388
Male [*n* (%)]	1 (6.67)	2 (7.69)	7 (30.43)	0.093
Proteinuria [*n* (%)]	NA	2 (7.69)	22 (95.65)	<0.001***
Hematuria [*n* (%)]	NA	4 (15.38)	16 (69.57)	<0.001***
Pyuria [*n* (%)]	NA	4 (15.38)	15 (65.22)	<0.001***
Cylindruria [*n* (%)]	NA	0 (0)	4 (15.38)	0.09
24 h proteinuria, median (IQR), mg/24 h	NA	223.8 (196.0–352.0)	3,210.0 (1,629.5–7,693.5)	<0.001***
ACR, median (IQR), mg/g	NA	13.8 (7.8–36.8)	2,987.1 (929.6–4,543.5)	<0.001***
WBC, median (IQR), × 10^9^/L	NA	5.0 (3.9–6.5)	5.6 (4.2–8.3)	0.118
Lymphocytes, (IQR), × 10^9^/L	NA	1.0 (0.5–1.4)	1.0 (0.6–1.8)	0.794
Hb, median (IQR), g/L	NA	99.0 (90.0–120.0)	96.0 (83.0–111.0)	0.411
PLT, median (IQR), × 10^9^/L	NA	169.0 (117.8–252.3)	182.0 (140.0–316.0)	0.293
ESR, median (IQR), mm/h	NA	37.0 (23.3–65.3)	51.9 (24.0–83.5)	0.176
Blood albumin, median (IQR), g/L	NA	36.1 (30.9–38.5)	30.5 (24.4–33.7)	0.002**
eGFR, median (IQR), ml/min/1.73 m^2^	NA	156.8 (126.5–185.8)	67.2 (28.8–139.0)	<0.001***
C3, median (IQR), g/L	NA	0.67 (0.46–0.88)	0.61 (0.52–0.87)	0.901
C4, median (IQR), g/L	NA	0.08 (0.03–0.17)	0.08 (0.04–0.3)	0.508
CD3^+^ T-cells (IQR), × 10^9^/L	NA	0.57 (0.33–0.75)	0.78 (0.41–1.44)	0.066
CD3^+^CD4^+^ T-cells (IQR), × 10^9^/L	NA	0.19 (0.13–0.38)	0.30 (0.15–0.57)	0.229
CD3^+^CD8^+^ T-cells (IQR), × 10^9^/L	NA	0.29 (0.16–0.39)	0.43 (0.23–0.85)	0.099
B cells (IQR), × 10^9^/L	NA	0.08 (0.03–0.23)	0.11 (0.05–0.23)	0.54
NK cells (IQR), × 10^9^/L	NA	0.05 (0.03–0.08)	0.11 (0.05–0.20)	0.015*
Th/Ts, median (IQR)	NA	0.63 (0.48–1.31)	0.69 (0.48–1.05)	0.67
anti-dsDNA, median (IQR)	NA	275.68 (43.99–654.44)	197.07 (90.09–476.17)	0.702
ANA [n (%)]	NA	20 (76.92)	16 (69.57)	0.56
25-(OH) D3, median (IQR), ng/mL	NA	14.00 (10.91–18.66)	11.33 (7.96–19.07)	0.268
SLE-DAI, median (IQR)	NA	4.0 (2.0–5.3)	12.0 (8.0–16.0)	<0.001***

***p < 0.001; **p < 0.01; *p < 0.05.

### 2.2. Comparison of the fungi diversity between SLE patients and healthy control

**FIGURE 1:**
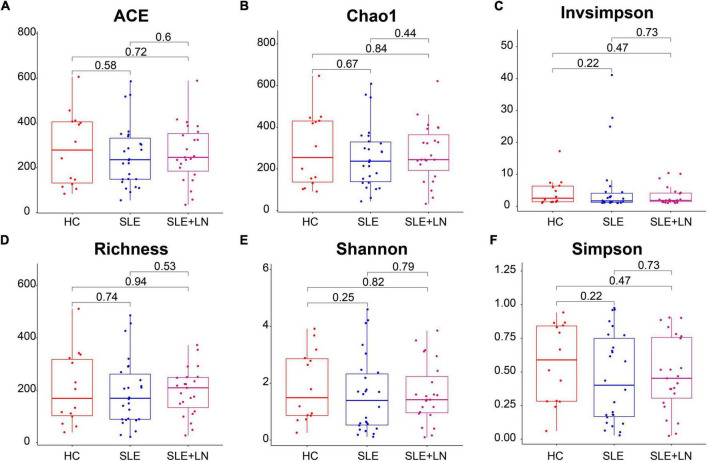
Comparison of the gut fungal diversity among SLE with LN, SLE without LN and HC groups. ACE **(A)**, Chao1 **(B)**, Invsimpson **(C)**, Richness **(D)**, Shannon **(E)**, and Simpson **(F)** describe the alpha diversity of the fungi in SLE and HC groups. HC, healthy control; SLE, SLE without LN; SLE + LN, SLE with LN.

**FIGURE 2:**
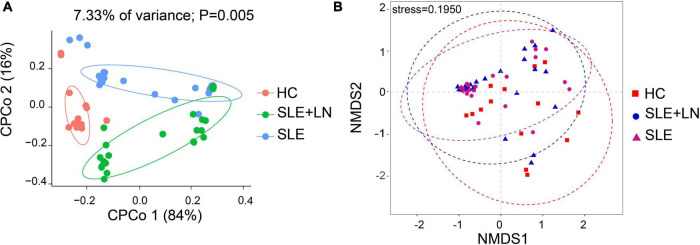
Changes of the fungal composition. **(A)** Constrained principal coordinate analysis of Bray–Curtis distance with each sample colored according to different groups. **(B)** Non-metric multidimensional scaling (NMDS) analysis of each sample colored according to different groups. HC, healthy control; SLE, SLE without LN; SLE + LN, SLE with LN.

To compare the fungal microbiota composition in the HC, SLE without LN, and SLE with LN groups, ITS1 gene sequencing on the feces was accomplished. More than 4.6 million tags were produced by the sequencing. After trimming and filtering, the predominant tag length seen among 0–260 bp (Supplementary Figure 1). For further analysis, we used Usearch high-quality sequences for clustering or noise reduction to obtain several clusters. The same or similar sequences were classified into the same cluster to obtain operational taxonomic units (OTUs) or amplicon sequence variants (ASVs), and then several clusters were obtained, called as Features. A total of 63 samples produced 4,507 Features, leaving 4,495 to be flattened. The final tags of each sample were 13,922. To assess the sequencing depth of all groups, which was larger than 0.990, the goods coverage index was chosen. Our sequencing depth and coverage were reasonable, as evidenced by the plateau rarefaction curves in both the SLE and HC samples, and this suggests that further data will only add a limited number of new features (Supplementary Figure 2). We looked into the variety within groups firstly. The measure of genus richness known as “alpha diversity” was assessed by ACE, Chao1, Invsimpson, Richness, Shannon, as well as Simpson. Results in Figures 1A–F indicated no apparent difference in alpha diversity between SLE and the healthy groups (*p* > 0.05). The CPCoA showed that individuals belonging to SLE with or without LN groups were fairly well-separated from the HC group. There is a slight overlap in SLE with or without LN (Figure 2A). In addition, the non-parametric multidimensional scaling (NMDS) was employed to determine the samples’ spatial placement within and between categories (Figure 2B), depicting the difference between the SLE with or without LN groups and the HC group. Meanwhile, there were significantly distinct groups between SLE and HC groups (Supplementary Figures 3A, B). Taken together, these results suggest that the patients with SLE exhibited a more unique fungal phenomenon compared to the healthy individuals.

### 2.3. The SLE fungal profile differs in HC patients

**FIGURE 3:**
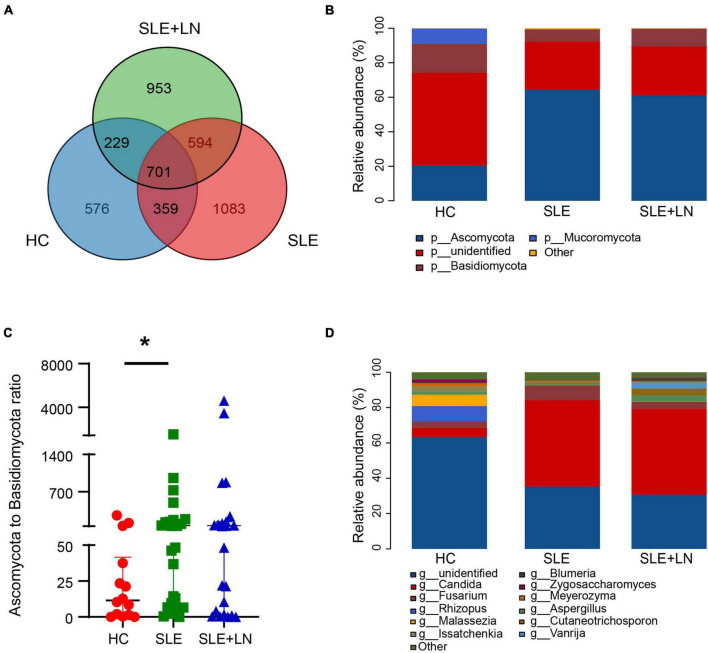
Diversity of the fungal composition. **(A)** Venn diagram analysis according to the amplicon sequence variants abundance among the three groups. **(B)** Comparisons of the relative abundance of dominant fungal taxa at the phylum level among groups. **(C)** The ratio of *Ascomycota* to *Basidiomycota* relative abundance among three groups. **(D)** Comparisons of the relative abundance of dominant fungal taxa at the genus level of all samples. *p < 0.05.

The three groups shared 701 OTUs, whereas the HC, SLE, and LN groups each had 576, 1,083, and 953 OTUs that were distinct from the others (Figure 3A). The counts of OTUs in the SLE with LN or without LN groups were higher than the HC group, revealing the different fungal characteristics of the SLE groups from those of HC group. We used the Unite database to compare the representative sequences of OTUs in order to gather taxonomic data for the OTUs at the phylum and genus levels. In terms of phyla, for the distribution of fungal taxa, in the HC group, the unidentified portion occupies the largest proportion of 53.7%, followed by *Ascomycota* (20.5%) and *Basidiomycota* (16.8%). While, in SLE with LN or without LN groups, *Ascomycota* was 64.5% and 61.0%, respectively, followed by the unidentified portion of 27.9% and 28.4% while *Basidiomycota* of 6.9% and 10.3% (Figure 3B). Moreover, the ratio of *Ascomycota*/*Basidiomycota* reflecting fungal ecological imbalance (Coker et al., 2019) was higher in SLE with LN or without LN groups than in HC group (Figure 3C). Reduced taxonomic levels revealed additional differences; in SLE with LN or without LN groups compared to the HC group, a significantly higher abundance of *Candida* and a significantly lower tendency of *Rhizopus* and *Malassezia* were discovered. The HC group gut fungi were composed of *Rhizopus* (8.8%), *Malassezia* (6.4%), *Candida* (5.3%), and the unidentified fungal genus contributed to the vast majority (63.3%) (Figure 3D). This finding is in line with earlier research, which revealed that the most common fungal genus in healthy people are *Saccharomyces* and *Cladosporium* (Li et al., 2018; Hu et al., 2022). Interestingly, *Candida* was found to be the dominant genus of the SLE without LN group (48.9%) and SLE with LN group (48.6%), followed by unidentified fungal genus of 35.3% and 30.7% (Figure 3D). Overall, these data elucidate the gut microbiota dysbiosis in patients with SLE, which could play a part in the onset and development of SLE.

### 2.4. Specific taxonomic changes

**FIGURE 4:**
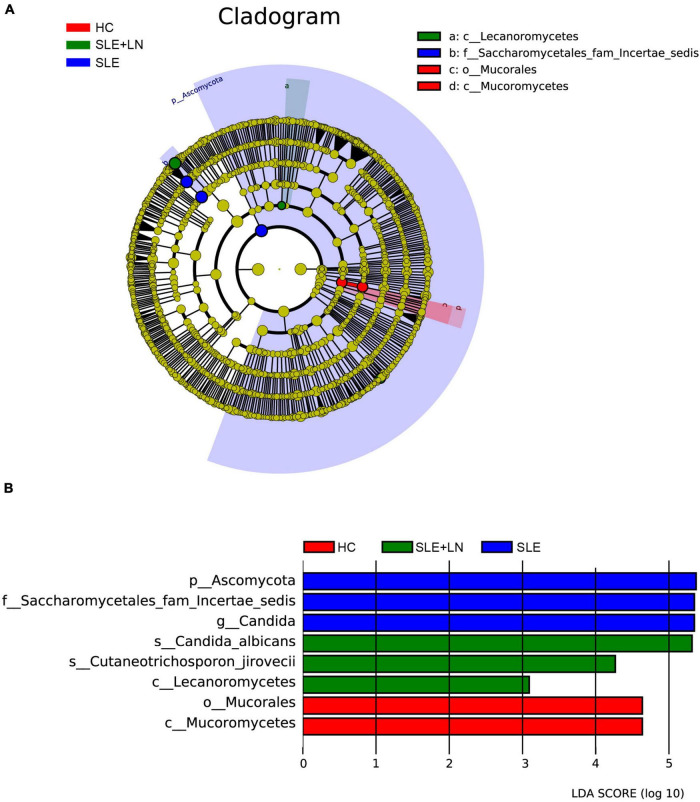
Fungal species as SLE diagnostic markers. **(A)** Cladogram results of the different fungal taxa. Differences were represented by the color of the most abundant class. The diameter of each circle is proportional to the abundance of taxa. Each ring represents the next lower taxonomic level. **(B)** LDA score computed by LEfSe analysis among HC group, SLE without LN group and SLE with LN group.

**FIGURE 5:**
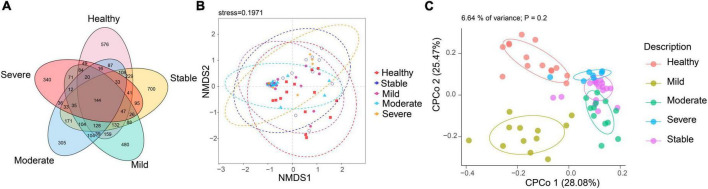
The altered microbial community compositions according to the SLEDAI score 2,000. **(A)** Venn diagram analysis among the five groups. **(B)** Non-metric multidimensional scaling (NMDS) analysis and **(C)** constrained principal coordinate analysis of Bray–Curtis distance with each sample colored according to different groups.

**FIGURE 6:**
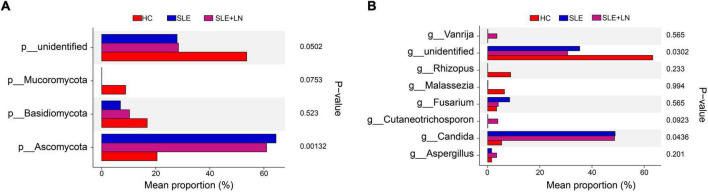
STAMP (statistical analysis of taxonomic and functional profiles) analysis of fungi microbiota. **(A)** STAMP analysis of fungi microbiota at the phylum level among groups. **(B)** STAMP analysis of fungi microbiota at the genus level among groups.

**FIGURE 7:**
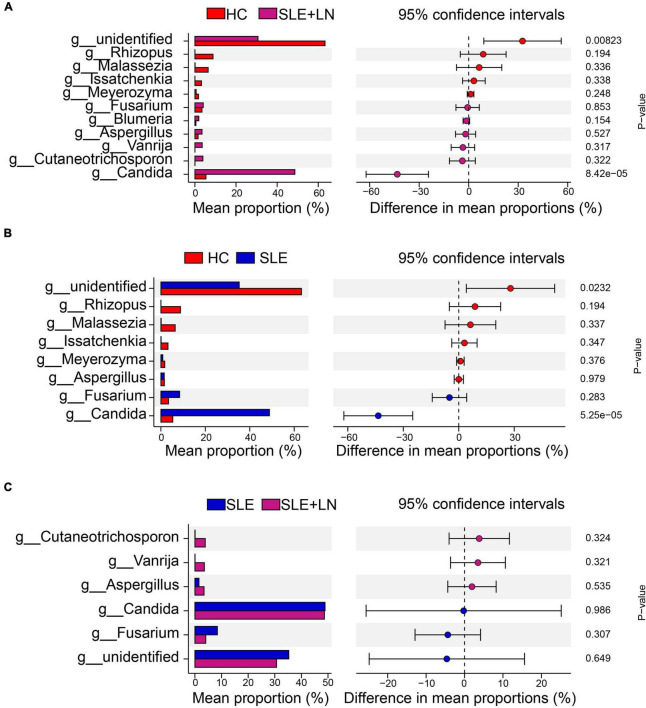
STAMP analysis based on two groups of *t*-test statistical tests. **(A)** STAMP analysis based on HC group and LN group *t*-test statistical tests. **(B)** STAMP analysis based on HC group and SLE group *t*-test statistical tests. **(C)** STAMP analysis based on the SLE and LN groups of *t*-test.

Linear discriminant analysis effect size (LefSe) analysis was used throughout taxa (Figures 4A, B) to further characterize the intergroup differences of fungi microbiota in SLE. In contrast, the Cladogram displayed a total of 8 differential taxa, including 2 classes, 2 species, 1 phylum, 1 order, 1 family, and 1 genus, which was identified as the candidate markers for discriminating between SLE and HC groups (LEfSe: *p* < 0.05, *q* < 0.05, LDA > 3.0). At various degrees, the prevalence of *Candida* was widespread throughout the SLE with and without the LN group. Other taxa’s specifics are displayed in Figures 4A, B. We defined the patients according to the SLEDAI score 2,000, and 0 to 4 were divided into the stable group, 5 to 9 for the mild activity group, 10 to 14 for the moderate activity group, and more than 15 as the severe activity group. Venn diagram shows that 144 OTUs were shared among the five groups, with 576,700, 480, 305, and 340 OTUs unique for the HC, stable, mild, moderate, and severe groups, respectively (Figure 5A). Among patients with SLE, there were no significant differences in Chao1, ACE, Richness, Shannon, Simpson, and Invsimpson estimates of alpha diversity with SLEDAI score (Mann–Whitney, *p* > 0.05). The microbial community constituents among the five groups were subsequently assessed by β diversity analysis of NMDS (Figure 5B) and CPCoA (Figure 5C). These results indicate the shifts in fungi taxonomic abundance in patients with SLE according to the SLEDAI score. Additionally, STAMP (statistical analysis of taxonomic and functional profiles) analysis of fungal microbiota showed that *Ascomycota* at the phylum level and *Candida* at the genus level were statistically significant among the three groups (Figures 6A, B) (*p* < 0.05). Further pairwise comparison between groups revealed that *Candida* was significantly different between HC and SLE groups, SLE with LN and without LN, however, did not significantly differ from one another (Figures 7A–C).

### 2.5. Mean decrease accuracy of top 20 fungi and random forest prediction model

**FIGURE 8:**
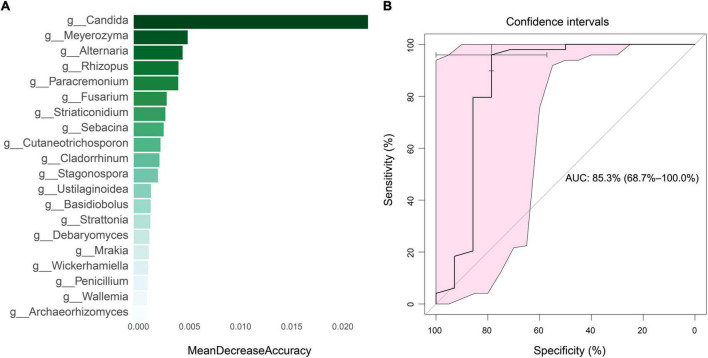
Mean decrease accuracy of top 20 fungi and diagnostic efficiency analysis. **(A)** Mean decrease accuracy of top 20 fungi between SLE and HC groups. **(B)** The diagnostic efficiency analysis of top five fungi based on random forest algorithm.

In order to identify potential diagnostic biomarkers of SLE, we used the random forest to establish a prediction model at the genus level. We performed a mean decrease accuracy analysis on the top 20 different fungi. *Candida* was the most important at the genus level, followed by *Meyerozyma*, *Alternaria*, *Rhizopus* and *Paracremonium* (Figure 8A). The model training randomly selected 70% of all samples as the training set and 30% as the test set, and obtained an area under the ROC curve of 85.3 (Figure 8B).

### 2.6. Functional classification prediction of the specific taxonomic

**FIGURE 9:**
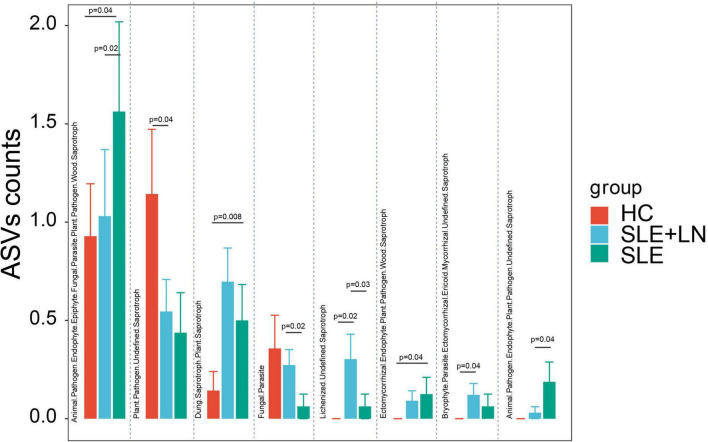
Fungal function classification prediction. Fungal functional annotations of the three groups were performed by FUNGuild. According to the absorption and utilization of environmental resources, fungi were divided into different categories. *p < 0.05.

The functional classification of the particular fungal population was further predicted using FUNGuild. We took the union of the different categories from the three groups and created a visualization that annotated the degree of differences between them. Compared with the HC group, the SLE group exhibited significant differences in several categories, including the increase in Animal Pathogen-Endophyte-Epiphyte-Fungal Parasite-Plant Pathogen-Wood *Saprotroph*, Dung *Saprotroph*-Plant *Saprotroph* and Ectomycorrhizal-Endophyte-Plant Pathogen-Wood *Saprotroph*, and a decrease in Plant Pathogen-Undefined *Saprotroph*. Similarly, compared with the HC group, the LN group showed a significant increase in Lichenized-Undefined *Saprotroph* and Bryophyte Parasite-Ectomycorrhizal-Ericoid Mycorrhizal-Undefined *Saprotroph*. The LN group exhibited significant differences in several categories, including an increase in Fungal Parasite and Lichenized-Undefined *Saprotroph*, as well as a decrease in Animal Pathogen-Endophyte-Epiphyte-Fungal Parasite-Plant Pathogen-Wood *Saprotroph*, Ectomycorrhizal-Endophyte-Plant Pathogen-Wood *Saprotroph*, and Animal Pathogen-Endophyte-Plant Pathogen-Undefined *Saprotroph* compared with the SLE group (Figure 9). There were distinctively differential functions among the three groups. The aforementioned findings thus point to a special ecological interaction that is necessary for preserving the homeostasis of SLE during the incidence and development of the disease.

### 2.7. Isolation and identification of fecal fungi

Based on the results of the ITS sequencing, we randomly selected a high-abundance fecal sample from the patients with SLE to further confirm the presence of fungi in the feces. First, it was inoculated on the chromagar plate, then cultured at 37°C in an incubator until it developed fungal colonies (Supplementary Figure 4A). Then, we picked fungal colonies on the plate to observe the morphological characteristics of the spores and hyphae (Supplementary Figure 4B) under a microscope. We used flight mass spectrometry to analyze the isolated colony and identified it as *Candida glabrata* strain according to the VITEK MS standard operating procedure (Supplementary Figure 4C).

### 2.8. Intestinal fungi correlate with clinical parameters in SLE

**FIGURE 10:**
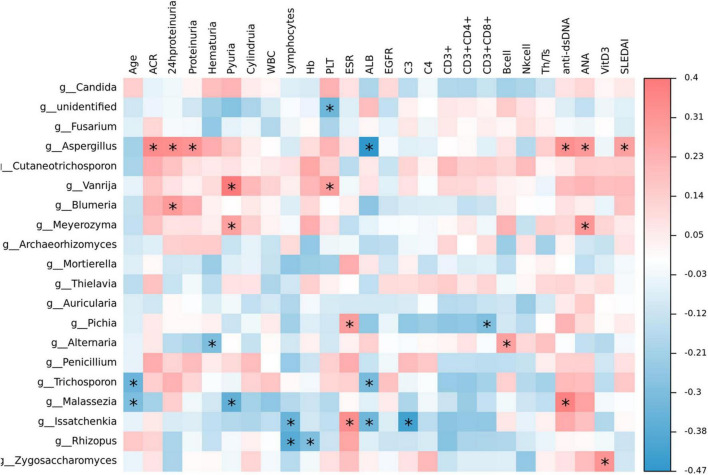
Correlation analysis. The heat map analysis showed a spearman association of clinical parameters in the SLE with LN group and SLE without LN group of the most abundant fungi at the genus level. *p < 0.05.

The heat map visualization displayed the correlation of clinical parameters with the SLE with LN or without LN group, of the most abundant fungi at the genus level and diversity (Figure 10). In SLE patients, *Aspergillus* was linked positively with ACR, 24 h proteinuria, proteinuria, anti-dsDNA, ANA, and SLEDAI, while *Issatchenkia* was positively linked with PLT but correlated negatively with lymphocytes, Alb and C3, respectively. Additionally, *Malassezia* was positively linked with anti-dsDNA but linked negatively with age and pyuria.

## 3. Discussion

The mammal body is home to trillions of microorganisms, including parasites, fungus, viruses, bacteria, and archaea (Li et al., 2018). In addition to bacteria and viruses, fungi are a minor yet significant component of the human gastrointestinal system. It makes up 0.1 percent of all gut microorganisms and is crucial for maintaining human intestinal homeostasis and regulating pathological situations (Sender et al., 2016; Nash et al., 2017). Over the past 10 years, sequencing technology has continued to advance (Hoggard et al., 2018) a large number of fungi in the intestine that are protective or harmful to human health are gradually discovered and verified. Fungal microbiota dysbiosis was considered to be associated with many diseases, including cancers (Conche and Greten, 2018; Wang et al., 2018; Coker et al., 2019; Dohlman et al., 2022; Lin et al., 2022; Narunsky-Haziza et al., 2022), autoimmune diseases (Lemoinne et al., 2020; Shah et al., 2021; Huo et al., 2022; Iliev, 2022), metabolic disorders (Sun et al., 2021), and chronic disease (Hu et al., 2022). The colonization and growth of opportunistic fungal pathogens in the intestine can induce host immune dysregulation (Limon et al., 2019; Jain et al., 2021; Zhang et al., 2022). It is mostly unknown how the gut’s fungal microbiomes differ in SLE. In this study, we used ITS1 sequencing to first pinpoint the differences in fungal composition and ecology across the three groups. Our findings revealed relationships between fungus species and clinical parameters and explained the changes in the fungus microbiome in SLE for the first time. This study serves as a crucial resource for comprehending how gut fungi contribute to SLE.

The etiologic mechanism of SLE remains not fully understood, although multiple associations have been identified due to decades of research. Similar to the studies of bacterial profiles in SLE patients (He et al., 2020; Chen B. et al., 2021; Shirakashi et al., 2022), we currently characterized the features of fungal microecological changes and revealed the alteration fluctuated in different stages of SLE. The SLE patients with LN or without LN showed differences in their gut fungal microbiome from that of the HC group. Additionally, compared to healthy participants, the fungal community differed in SLE patients in the stable phase, mild activity, moderate activity, and severe activity. These findings are useful additions to the earlier research on the bacterial microbiome in SLE patients at various disease phases (Azzouz et al., 2019). Additionally, we demonstrated for the first time that individuals with SLE had a gut fungal dysbiosis, which is defined by a relative decline in biodiversity and an altered composition.

We also identified the changes in SLE-specific fungal composition, including the obvious enrichment of *Candida* of *Ascomycota* phylum and the loss of *Malassezia* of *Basidiomycota* phylum, implying their potential role in the pathogenesis of SLE and deserving further investigation. *Candida* is an important opportunistic pathogenic fungus (Witchley et al., 2019) and human symbiotic fungi in the gastrointestinal tract (Alonso-Monge et al., 2021; McDonough et al., 2021), oral cavity (Millet et al., 2020; Lemberg et al., 2022), and vagina (Jang et al., 2019; Alvendal et al., 2020). The excessive growth of *Candida* is associated with a variety of diseases, including thrush (Salvatori et al., 2016) and vaginal candidiasis. Recent research has also discovered that *Candida* is connected in a close way to inflammatory bowel disease (Li et al., 2022) and gastrointestinal cancer (Zhong et al., 2021). In this study, we identified previously unreported SLE-associated gut fungi, such as *Candida glabrata* and *Malassezia*, that could be a factor in the development of SLE. We also evaluated the diagnostic model of SLE-associated fungi in distinguishing SLE from HC groups, with the area under the curve (AUC) of 0.853 (95% CI: 0.687-1). Additionally, *Malassezia* usually exists on the skin (Sparber et al., 2019) and can colonize in the gastrointestinal tract (Aykut et al., 2019; Li et al., 2022), particularly in the elderly adults (Shuai et al., 2022). Interestingly, our data indicate an obvious downregulation of *Malassezia* in patients with SLE, most probably it would be inhibited by *Candida albicans* according to the view that the dominant flora inhibits the growth of other flora (Lu and Stappenbeck, 2022). These results provide ideas for the application of unique fungal markers to detect and monitor the prognosis of SLE. Undoubtedly, further studies with large sample sizes and strict screening standards, along with multicenter joint projects are recommended for the verification of these findings to be used widely in future.

**FIGURE 11:**
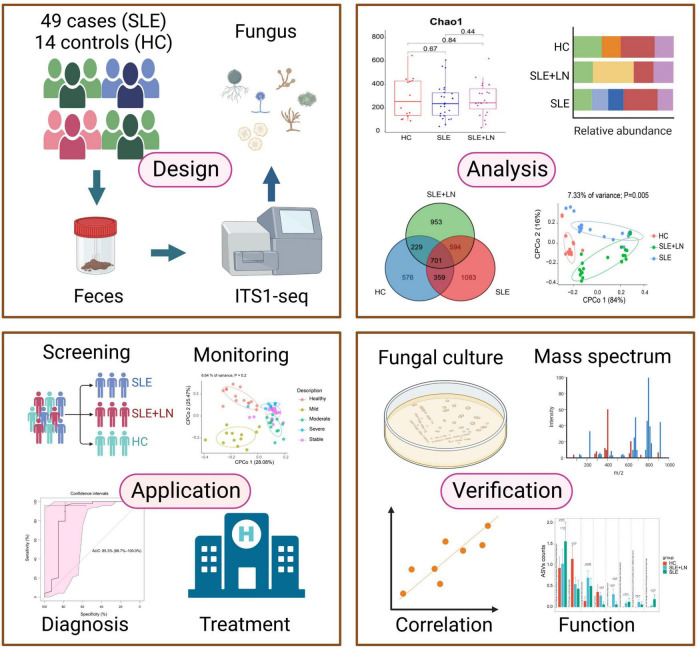
This flowchart illustrates the conceptual framework for the proposed study. Created by BioRender.com.

In addition, our study showed that intestinal fecal fungal markers might be involved in the SLE pathology. The difference was identified in SLE-unique fungi and clinical parameters, indicating that host-fungal synergistic interactions may perform a part in the development of SLE. It has been established that intestinal fungi have a role in the regulation of physiological processes, host pathology, homoeostasis, as well as in the development of the co-existing gut bacterial microbiome (Zhang et al., 2022). To sum up, our study has gone through four phases (Figure 11). Briefly, by performing ITS1 sequencing on the collected fecal samples, we obtained fungi that colonized during SLE progression, with the changes in biodiversity and microbial composition. In the validation phases, we verified the presence of fungi by inoculating chromagar plate, microscope examination and mass spectrometry. Finally, we assessed the diagnostic ability of the top 20 different fungi and constructed predictive models for random forest prediction analysis of the five most important fungi to identify potential diagnostic biomarkers which may be used for diagnosis with a certain degree of accuracy in the future.

However, the ecological effect between fungi and bacteria within the gut, and their potential interaction influencing the host immune homeostasis are fascinating and confusing (Richard and Sokol, 2019; Ren et al., 2022). In order to fully understand the diagnostic and therapeutic targets against SLE, further multi-omics studies on the interactions between bacteria, fungi, virus, and the host are needed (Richard and Sokol, 2019; Lam et al., 2022; Liu et al., 2022).

In conclusion, our research demonstrated the distinctive mycobiome of the gut and highlighted the possible significance of the gut mycobiome in SLE. Our results offer a crucial resource and can be a useful addition when developing novel SLE diagnostic and treatment approaches. Future research is necessary to confirm the purpose and mechanism of the alternating gut fungal composition and ecology in order to better understand the role of the gut fungal microbiome in the incidence and progression of SLE. We subsequently considered to isolate the fungi and verify it on humanized SLE mice to explore its role in the occurrence and development of SLE. Undeniably, because of the limited sample size, high scale of unclassified taxa, and single-center status of our study, randomized clinical trials with large cohort in multi-centers, and more advanced sequencing technology should be considered in future to further validate our findings.

## 4. Materials and methods

### 4.1. Design of the study

We recruited 49 SLE patients consisting of 26 SLE patients without LN and 23 SLE patients with LN which satisfied the revised criteria for SLE established by the American College of Rheumatology in 1997 (ACR) (Hochberg, 1997; Petri et al., 2012) and 14 healthy controls from the Affiliated Drum Tower Hospital of Nanjing University Medical School in this study. The enrollment of LN was identified by rheumatologist according to the 2012 ACR guidelines for screening, treatment, and management of LN (Hahn et al., 2012). A total of 63 feces (49 SLE feces and 14 healthy feces) were collected for gut microbiota analysis when the patient has just been admitted to the hospital and has not been treated. SLE disease activity was estimated according to the SLEDAI score. Ethics Committee of the Affiliated Drum Tower Hospital of Nanjing University Medical School provided the approval for the study (ID:2022-466-01), and informed written consent was also obtained from each participant of the study. All feces were stored at −80°C for subsequent use. DNA was extracted by improved CTAB method according to the manufacturer’s guidelines (QIAGEN, Hilden, Germany). The extracted genomic DNA was tested by 1% agarose gel electrophoresis (Thermo Fisher Scientific, Wilmington, NC, USA). Depending on the concentration, DNA was diluted to l μg/μL using sterile water. ITS genes of distinct regions (ITS-5F) amplification were performed using the primers ITS5-1737F GGAAGTAAAAGTCGTAACAAGG; ITS1-2043R GCTGCGTTCTTCATCGATGC. NanoDrop 2000 UV-Vis spectrophotometer was used to measure the DNA content and cleanliness. The ITS1 rRNA PCR amplification was performed using the given protocol: All PCR reactions were performed using a 15 μL Phusion^®^ High-Fidelity PCR Master Mix (New England Biological Laboratory), 0.2 μM of forward and reverse primers and approximately 10 ng template DNA. Initial denaturation at 98°C for 1 min, followed by 30 cycles of 10 s at 98°C, 30 s of annealing at 50°C, and 30 s of elongation at 72°C, then single extension at 72°C for 5 min, and 10°C until stopped.

The PCR products detected by electrophoresis on 1% agarose gel was examined by electrophoresis and purified using Agencourt AMPure XP Nucleic Acid Purification Kit. On manufacturer’s recommendation, a sequencing library was generated using the TruSeq^®^ DNA PCR Sample-Free Preparation Kit (Illumina, USA) with an index code added. The quality of the library was evaluated on the Qubit@2.0 Fluorometer (Thermo Scientific) and Agilent Bioanalyzer 2100 system. On the Illumina NovaSeq platform, extracted amplicons were aggregated in an equimolar ratio and paired-for-end sequencing (Illumina, San Diego, CA, USA). The raw reads were uploaded to the Sequence Read Archive (SRA) database at the National Center for Biotechnology Information (NCBI) (Accession Code: PRJNA900285).

### 4.2. Processing of sequencing results and taxonomical annotation

Raw data were first filtered and eliminated from consideration the sequence that were shorter than 230 bp, had a low-quality score (≤20), contains ambiguous bases or did not exactly match the primer sequence and the barcode tags, and was separated using a sample-specific barcode sequence. Then the high-quality sequences were de-noised using the Unoise3 method by usearch11. The Unoise3 denoising sequences are often referred to as an amplicon sequence variant (ASVs). According to the Unite database, the BLAST tool was used to classify all sequences into different taxonomic groups. The study of bar-plot diagrams was done using R (v3.6.0) program based on the findings of taxonomic annotation and relative abundance. Relying on the Unite database, the BLAST tool was used to classify all sequences into different classification groups. R (v3.6.0) software was employed for bar chart analysis based on the results of classification annotation and relative abundance.

### 4.3. Statistical analysis

Quantitative Insights Into Microbial Ecology [QIIME (v1.8.0)] was used to generate rarefaction curves, and calculated the observed species and chao1 indices based on the ASV information. In order to test the similarity between different samples, R (v3.6.0) was used to analyze index of observed species and chao1 and PCoA based on the ASV information of each feces sample. Linear discriminant analysis (LDA) effect size (LEfSe) (Segata et al., 2011) was done to find new ideal fungal markers. Through the Kruskal–Wallis rank sum test, we can obtain different feature or species. Statistical analyses were performed using an Statistical Product and Service Solutions (SPSS 24.0) (SPSS Inc., Chicago, IL, USA), and GraphPad Prism 8.0 (San Diego, CA, USA). *p* < 0.05 represents a statistically significant difference. Wilcoxon rank test and Turkey group test were performed by the R project. The FUNGuild (version 1.0) was used to infer the prediction of fungal functional classification (Nguyen et al., 2016). Python was used to calculate the Gini index and ggplot was used to display it.

## Data availability statement

The datasets presented in this study can be found in online repositories. The names of the repository/repositories and accession number(s) can be found below: https://www.ncbi.nlm.nih.gov/, PRJNA900285.

## Ethics statement

The studies involving human participants were reviewed and approved by 2022-466-01. The patients/participants provided their written informed consent to participate in this study.

## Author contributions

PY and ZX: methodology, data validation, and writing—original draft. PY: formal analysis. FC and SC: sample collection and clinical information processing. RX: investigation and data curation. AK: methodology and language editing. SC, LL, and XZ: partial picture drawing. ZX, YW, and HS: conceptualization and supervision. All authors contributed to the article and approved the submitted version.
