# Supplementary material for: Fungal gut microbiota dysbiosis in systemic lupus erythematosus
Source: Front Microbiol. 2023 Apr 5;14:1149311. doi: 10.3389/fmicb.2023.1149311 (PMC10115219; doi:10.3389/fmicb.2023.1149311)
Supplement: Supplementary file 1 [file Data_Sheet_1.docx]

Supplementary Material

Fungal Gut Microbiota Dysbiosis in Systemic lupus erythematosus

*** Correspondence:** [ybwang@nju.edu.cn](mailto:ybwang@nju.edu.cn);[zhipengxu@njmu.edu.cn](mailto:zhipengxu@njmu.edu.cn); shenhan@njglyy.com

Supplementary Data

# Supplementary Figures


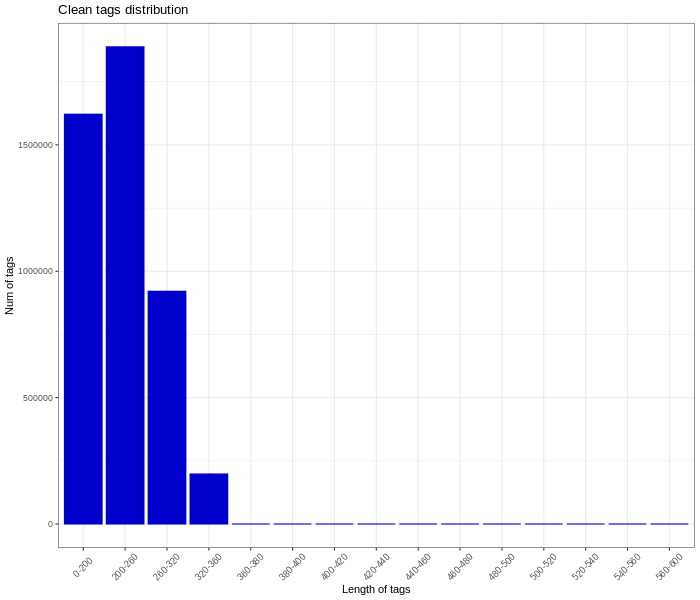


**Supplementary Figure S1. Clean Tags Distribution of all the sample.**

**
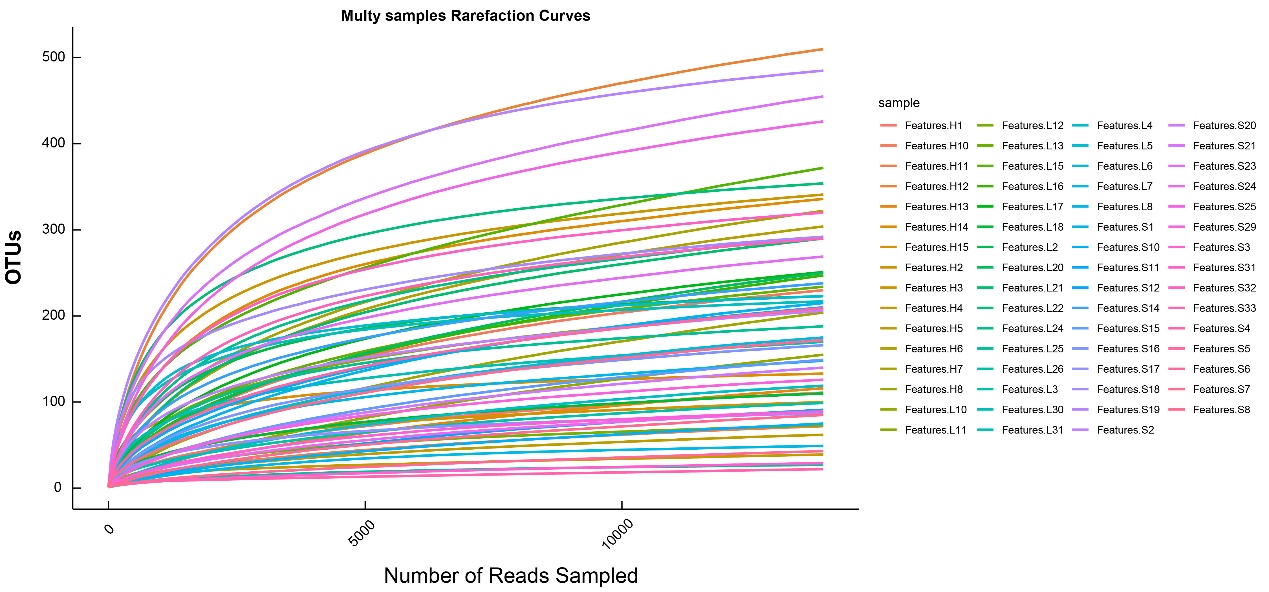
**

**Supplementary Figure S2. Rarefaction Curves showing the adequate depth of the sequencing of all samples.**

**
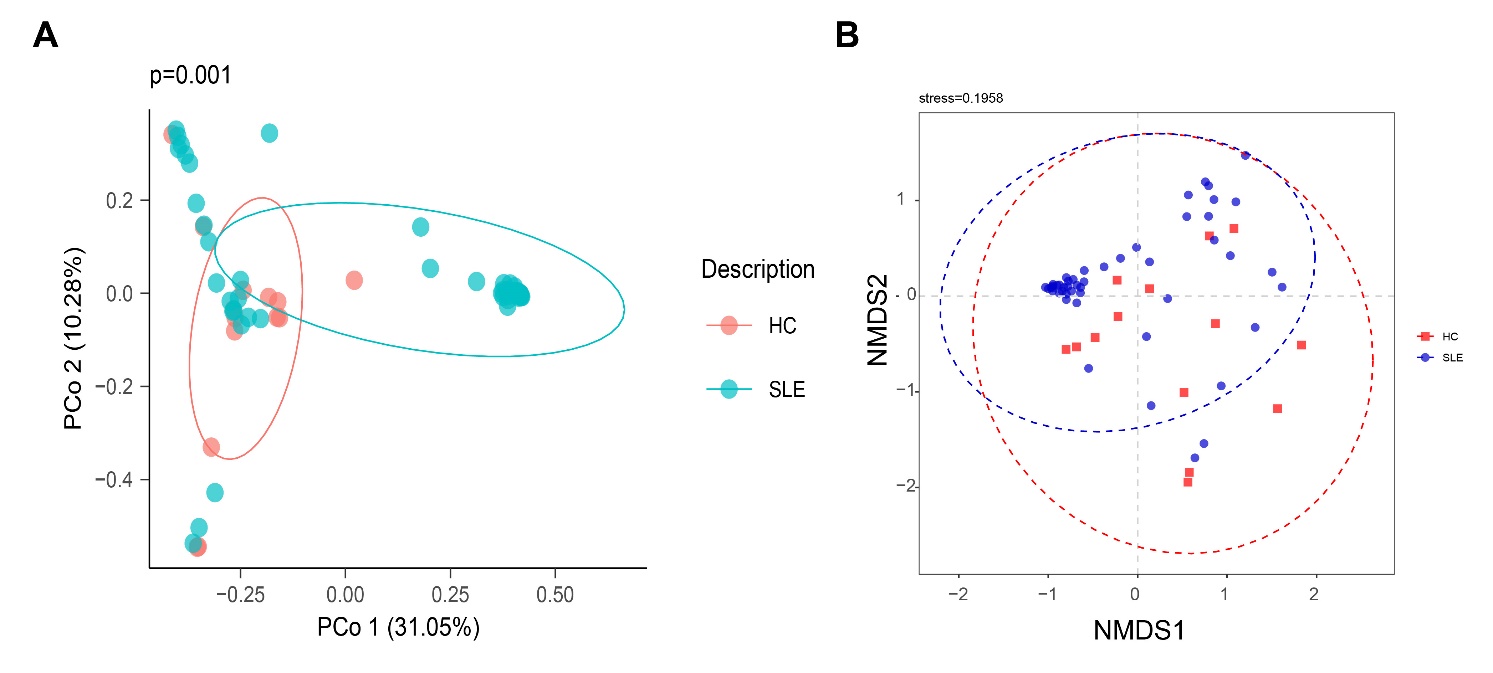
**

**Supplementary Figure S3. Changes of the fungal composition.** (A) Constrained Principal coordinate analysis of Bray–Curtis distance with each sample colored according to different groups. (B) Non-metric multidimensional scaling (NMDS) analysis of each sample colored according to different groups.

**
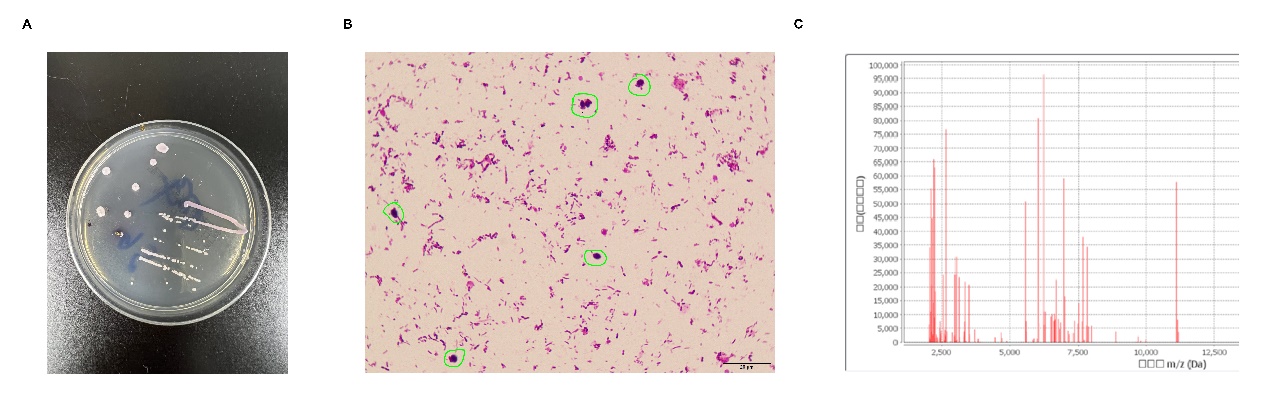
**

**Supplementary Figure S4. Isolation and Identification of Fecal Fungi.** (A) The chromagar plate of fungal colonies. (B) Morphological identification of fungi under oil lens. (The fungal spores were marked by the green circle). (C) Identification of isolated fungi by flight mass spectrometry.
